# Predicting Genetic Disorder and Types of Disorder Using Chain Classifier Approach

**DOI:** 10.3390/genes14010071

**Published:** 2022-12-26

**Authors:** Ali Raza, Furqan Rustam, Hafeez Ur Rehman Siddiqui, Isabel de la Torre Diez, Begoña Garcia-Zapirain, Ernesto Lee, Imran Ashraf

**Affiliations:** 1 Department of Computer Science, Khwaja Fareed University of Engineering and Information Technology, Rahim Yar Khan 64200, Pakistan; 2 School of Computer Science, University College Dublin, D04 V1W8 Dublin, Ireland; 3 Department of Signal Theory and Communications and Telematic Engineering, University of Valladolid, Paseo de Belén 15, 47011 Valladolid, Spain; 4 Department of Computer Science, Electronics and Telecommunications, University of Deusto, 48007 Bilbao, Spain; 5 College of Engineering and Technology, Miami Dade College, Miami, FL 33132, USA; 6 Department of Information and Communication Engineering, Yeungnam University, Gyeongsan 38541, Republic of Korea

**Keywords:** genome mutation, genetic disorder, machine learning, chain classifier approach

## Abstract

Genetic disorders are the result of mutation in the deoxyribonucleic acid (DNA) sequence which can be developed or inherited from parents. Such mutations may lead to fatal diseases such as Alzheimer’s, cancer, Hemochromatosis, etc. Recently, the use of artificial intelligence-based methods has shown superb success in the prediction and prognosis of different diseases. The potential of such methods can be utilized to predict genetic disorders at an early stage using the genome data for timely treatment. This study focuses on the multi-label multi-class problem and makes two major contributions to genetic disorder prediction. A novel feature engineering approach is proposed where the class probabilities from an extra tree (ET) and random forest (RF) are joined to make a feature set for model training. Secondly, the study utilizes the classifier chain approach where multiple classifiers are joined in a chain and the predictions from all the preceding classifiers are used by the conceding classifiers to make the final prediction. Because of the multi-label multi-class data, macro accuracy, Hamming loss, and *α*-evaluation score are used to evaluate the performance. Results suggest that extreme gradient boosting (XGB) produces the best scores with a 92% *α*-evaluation score and a 84% macro accuracy score. The performance of XGB is much better than state-of-the-art approaches, in terms of both performance and computational complexity.

## 1. Introduction

The genetic disorder is caused by a mutation in the genome or by a change in the gene structure [1]. As the genome carries the information, the change in the genome can result in a change in the structure or function of an organism [2]. The genes are made of deoxyribonucleic acid (DNA), and any change in the sequence of DNA results in genetic disorders. The genome data contains important information and health care indicators that can be used to analyze the genetic disorders that cause diseases. A dedicated branch of bioinformatics, genomics, focuses on the study of genomes, their structure, abnormalities, etc. [3]. There are several genetic disorders: single gene inheritance disorders [4], chromosomal disorders or mitochondrial genetic inheritance disorders [5], and complex disorders or multifactorial genetic inheritance disorders [6] and they are examined based on the DNA structure [7]. The single gene disorder type is caused by a mutation in a single gene in the DNA. The chromosomal disorder type is caused when a chromosome or a part of chromosomes is deleted or replaced in the DNA structure. Complex disorders are caused by the mutation in more than one gene present in the DNA.

The genes present in the DNA carry important information that can explain the formation of the different types of proteins [8]. Some changes in the structural properties of the gene can result in the formation of an abnormal protein. The abnormal protein does not work properly in the cell. Such abnormalities in the DNA gene lead to different genetic disorders such as cancer [9], diabetes, Alzheimer’s, etc. In 2020, 15,000 people were diagnosed with syndrome B disorder. About 100,000 children currently have syndrome B disorder while 12,000 people died from syndrome C disorder around the world [10]. Approximately 2% to 5% of all childbirths are diagnosed with genetic disorders [11] that may lead to 5% to 50% of deaths in the childhood [12]. The genome data contain useful information and several health-related indicators that can be used to predict the genetic disorder. However, keeping in view the complex nature of the DNA data, the number of features, and the volume of the data, manual prediction is laborious, error-prone, and inefficient.

Recently, the use of machine learning-based models has provided a great success in different fields, including prognosis, prediction, medicine, automation, etc. [13]. Such models are trained using good-quality historic data. A machine learning model finds the relationship or patterns in the data to make predictive decisions. Such models can provide automated prediction, as well as can perform assistive roles for medical experts, concerning the sensitivity and importance of the task. The choice of machine learning techniques is based on the type of real-world problem [14]. Machine learning techniques have a large variety of potential applications in bioinformatics [15]. The exponential growth of biological data raises the problem of management and useful information extraction. The transformation of heterogeneous data into biological knowledge is the main challenge in computational biology [16]. Machine learning models are applied to make a predictive decision based on large gene sequences and manage the large biological data. There are many biological domains currently using machine learning approaches for knowledge extraction. The applications of machine learning include analysis of genome-wide association [17], X-rays [18], enzyme function prediction [19], protein function prediction, and many more.

Machine learning models can help with precision medicine, however, often limited by low accuracy. Often using a single feature extraction approach, the sensitivity and specificity of the models are low than expected which requires further improvements. This study contributes to improving the predictive capabilities of machine learning models and makes the following key contributions:

- Genetic exploratory data analysis (GEDA) is performed to obtain useful insights and discover important information from the genome data. Various attributes and their distributions are investigated to analyze their trends regarding different disorders.
- A novel approach to extracting features from the genome data is designed where the extra tree (ET) and random forest (RF) are used to extract features that are combined to enrich the feature set.
- A chain classifier approach is adopted in this study to obtain higher prediction accuracy. Machine learning models, equal to the number of classes, are placed in the chain and each classifier predicts in the specified sequence. Each conceding model uses the predictions of all preceding models as the input to make its prediction.
- Eight machine learning models are also used for performance analysis including logistic regression (LR), multi-layer perceptron (MLP), decision tree classifier (DTC), random forest classifier (RFC), k nearest neighbors (KNN), extra tree classifier (ETC), extreme gradient boosting (XGB), and support vector classifier (SVC). Hyperparameter fine-tuning is completed for performance optimization. In addition, data balancing is applied to the genomes data to reduce the probability of model overfitting.
- Extensive experiments are performed to analyze precision, recall, accuracy, and F1 score. Moreover, performance comparison with existing studies is carried out in terms of training time, macro accuracy, Hamming loss, and *α* evaluation score.

The remainder of this article is structured as follows: the related literature is examined in Section 2. Section 3 contains a description of the methodology, multi-label multi-class chain classifier approach, and the proposed ETRF technique. Results and evaluations of the proposed approach are examined in Section 4. Finally, the conclusions are elaborated in Section 5.

## 2. Related Work

The related literature to our proposed research approach is examined in this section. The past applied techniques and proposed approaches are analyzed. The related literature analysis is based on the previous dataset used, limitations, applied approach, and performance results.

Alzheimer’s, one of the diseases caused by the genetic disorder has been investigated by several researchers [20]. For example, [21] presents a stacked machine learning model for Alzheimer’s prediction. The AD genetic dataset of the neuroimaging project [22] is utilized for experiments. Results suggest that the proposed stacked model can obtain an accuracy score of 93%. The research findings proved the effectiveness of stacked-based models for predicting Alzheimer’s disease. Similarly, classification of Alzheimer’s disease is performed using neuroimaging initiatives in [22]. Experiments are performed using the genetic dataset [23]. For this purpose, machine learning-based feature selection from the gene data is utilized. The age and number of education years features are added as additional features. The non-genetic factors are also considered for Alzheimer’s classification. The study proposes a novel XGBoost model for classification [24]. The proposed approach achieves 64% for the area under the curve (AUC).

The prediction of complex genes using supervised machine learning methods is carried out in [25]. The complex genes lead to a large number of diseases [26]. The GEO dataset is utilized for machine learning-based model testing. The study develops a machine learning-based genetic disease analyzer (GDA) using principal component analysis (PCA), Naive Bayes (NB), random forest (RF), and decision tree (DT) techniques. The proposed approach achieves a 98% of accuracy score.

The study [27] uses a supervised machine learning approach to predict dementia, cancer, and diabetes. The multifactorial genetic inheritance disorder multiclass dataset is used to perform experiments. The employed learning techniques are KNN and SVM where SVM proves to be superior with a 92% accuracy score. The inflammatory bowel disease prediction using machine learning techniques is proposed in the study [28]. The metagenomic dataset on inflammatory bowel disease multi-omics is utilized for machine learning model building and experimental research evaluations. Several machine learning classifiers are applied, and RF outperforms with a 90% accuracy score.

The study [29] uses machine learning techniques to predict COVID-19 infection and related diseases. The genetic SNP mutation dataset utilized RF and neural networks. RF shows superior performance with a 92% accuracy score. The prediction of familial hypercholesterolemia genetic disorder of lipid metabolism by using machine learning techniques is performed in [30]. The virtual genetic, clinical test of familial hypercholesterolemia is performed for experimental results evaluations. Of the machine learning models used for experiments, the gradient boosting classifier shows an 83% accuracy score. The study [31] proposes a machine learning-based algorithms DOMINO to predict dominant (monoallelic) mutations in genes for Mendelian disorders. The proposed DOMINO is based on the linear discriminant analysis. Experimental results reveal a 92% accuracy which is better than existing approaches.

In biomedical areas, gene-based disease prediction is a prominent issue and several researchers are working in this domain [32]. A machine learning-based model for the prediction of gene diseases is proposed in [33]. The study focuses on the binary class problem and classifies the disease genes and healthy genes. For experiments, 12 representative machine learning-based models are examined in terms of comparisons and interpretability. Table 1 contain the summary of related work.

The prediction of autism spectrum disorder from the genome data is investigated in [35]. A novel gene selection technique is utilized to find the candidate biomarker genes [38]. The phenotypic group associative genetic markers are utilized for the prediction task. The gene expression carries the specie genetic information and gene patterns show the relationship of genes associated with numerous diseases. The optimal features are identified by regularized genetic algorithms. The proposed approach achieves a 97% of accuracy score.

The Alzheimer’s disease prediction is carried out in [36] where a machine learning-based model is designed. Next-generation sequencing techniques are utilized for identifying biomarkers for diseases that help early diagnosis. The proposed method achieves an 81% accuracy score by using 10-fold cross-validation. A network-based technique named brainMI is proposed for brain disease gene prediction [37]. The predicting is performed by brain connectome integrating and molecular network. The brain connectome data are utilized for model building. The support vector machine model is utilized to predict gene brain diseases. The proposed model achieved a 72% accuracy score.

## 3. Methods

**Figure 1:**
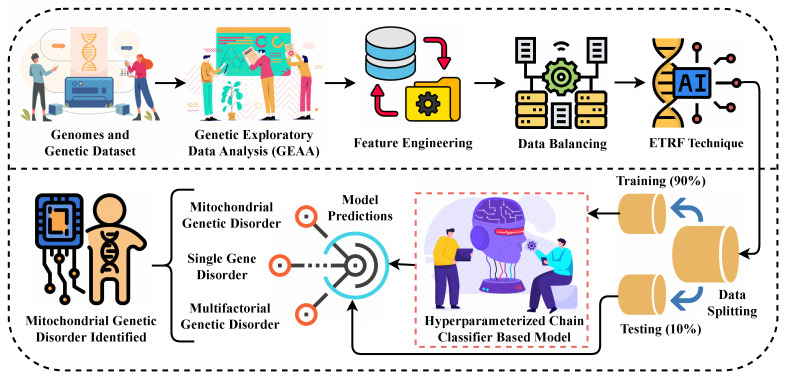
The methodological analysis of our proposed research approach for predicting the genetic disorder and types of disorder.

The multi-label multi-class genomes and genetics dataset is utilized for the proposed approach. Figure 1 shows the steps followed in the proposed approach. GEDA is applied to reveal the factors that cause genetic disorders and useful insights are obtained regarding genes. Feature engineering techniques are employed to feature data mapping and select the high-importance features to achieve better performance from the models. The data balancing of the genetic disorder class is applied to train the learning model on an equal number of data distributions which also helps to improve the performance. The novel ETRF feature extraction technique is applied to enrich the feature set which is later used for training all the models.

### 3.1. Genomes Dataset

The genome and genetic dataset are based on the medical information of children and adult patients who have genetic disorders [39]. The type of dataset is multi-label multi-class. The first attribute of the dataset is genetic ‘disorder’ and the second sub-label is ‘disorder subclass’. The dataset contains a total of 44 attributes. The dataset-related information is analyzed in Table 2.

### 3.2. Genetic Exploratory Data Analysis (GEDA)

GEDA is applied to the genomes dataset to find hidden patterns and important information that may be helpful to predict genetic disorders. GEAA is based on several graphs, such as pair plots, 3-D data distributions analysis, bar charts, and scatter plots. GEAA proves helpful in the research study to find statistical insights from the gene data.

**Figure 2:**
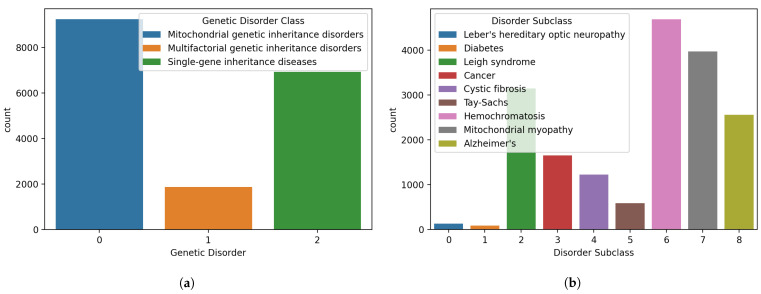
Distributions of samples for different classes in the dataset, (**a**) genetic disorders’ main classes, and (**b**) genetic disorder subclasses.

The genetic disorder label and the disorder sublabel data distribution analysis are applied and the results are visualized in Figure 2. The analysis shows that the dataset has an equal distribution. Genetic disorder attribute has three classes: single gene inheritance diseases, mitochondrial genetic inheritance disorders, and multifactorial genetic inheritance disorders. The mitochondrial genetic inheritance disorders class has the highest data distribution while the multifactorial genetic inheritance disorders have the lowest number of samples. The subclass category has nine classes: Leber’s hereditary optic neuropathy, diabetes, Leigh syndrome, cancer, cystic fibrosis, Tay-Sachs, hemochromatosis, mitochondrial myopathy, and Alzheimer’s. Leber’s hereditary optic neuropathy and diabetes have the lowest data distribution values. Similarly, the number of samples for Tay-Sachs is comparatively low.

**Figure 3:**
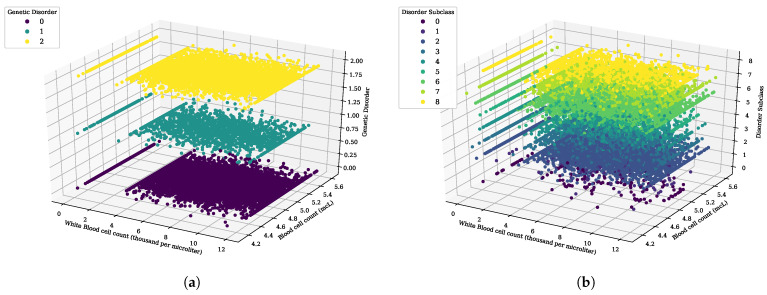
The 3D scatter analysis for white blood cell count (thousand per microliter) and blood cell count (mcL), (**a**) genetic disorder category, and (**b**) genetic disorder sub-category.

The 3-D scatter data distribution analysis of white blood cell count (thousand per microliter) and blood cell count (mcL) features of genomes data with the genetic disorder label is analyzed in Figure 3. The analysis demonstrates that when the white blood cell count is less than zero, a genetic disorder of all types is found. When the white blood cell count is between 0 and 2, there is no chance of type 0 (mitochondrial) genetic disorder. However, type 1 (multifactorial) and type 2 (single-gene) disorders are found in patients. Blood cell count (mcL) values of 4.2 or less show that there are no genetic disorders. White blood cell count (thousand per microliter) value from 2 to 12 and the blood cell count (mcL) values from 4.3 to 5.6 demonstrates that genetic disorders of all types are found in patients. This analysis provides us with the significant range values of blood cells that cause genetic disorder diseases.

Figure 3b shows the scatter plot for the distribution of blood cell count (mcL) and white blood cell count (thousand per microliter) with the disorder subclass. There is no subclass disease found when the blood cell count value ranges from 4.2 to 4.4. Leber’s hereditary optic neuropathy disease is found when the blood cell count value varies between 4.4 and 4.8. White blood cell count values of 0 to 2 show the lowest chances of genetic disorder subclass. A value above 4.8 for blood cell count demonstrates the occurrence of all subclass disorder diseases. This analysis provides the significant values of blood cells that are involved in sub-types of genetic disorders and diseases.

**Figure 4:**
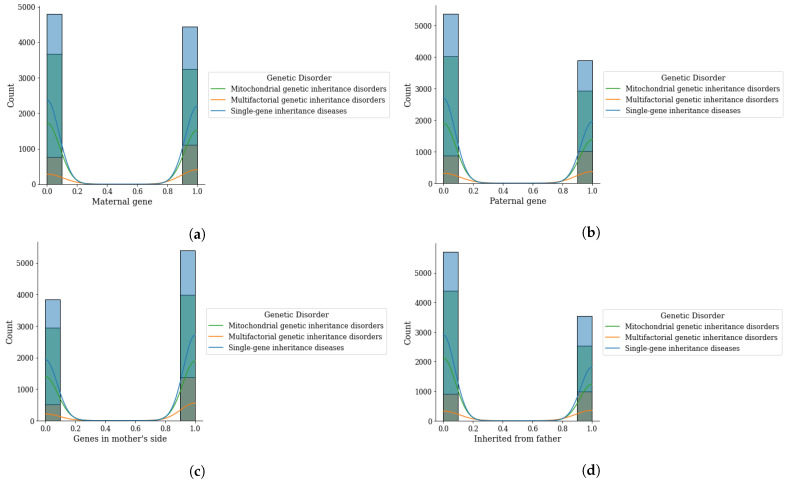
Genomes data distribution by genetic disorder category, (**a**) maternal gene, (**b**) paternal gene, (**c**) genes from mother side, and (**d**) inherited from father.

Analysis of inherited genes that cause the genetic disorder is given in Figure 4. The analyzed genes are based on the maternal gene, paternal gene, genes from the mother’s side, and inherited from the father. This analysis demonstrates that the genes are the prominent factors that are causing the genetic disorder. Analysis reveals that when the maternal and paternal gene value is 0 or 1 the mitochondrial disorder has a higher probability while single gene disorder has less chance of happening. Similarly, Figure 4c,d demonstrate that the mitochondrial disorder has a higher chance when the values of the genes on the mother’s side and inherited from the father are 0 or 1.

**Figure 5:**
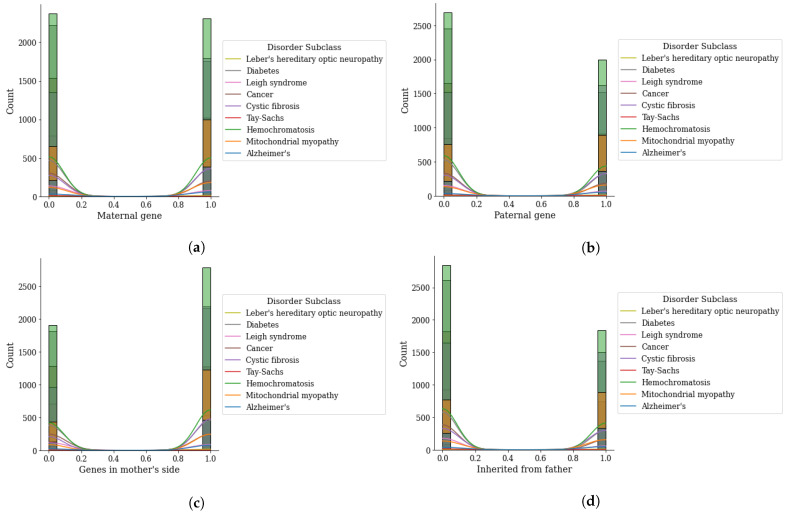
Genomes data distribution by genetic disorder sub-category, (**a**) maternal gene, (**b**) paternal gene, (**c**) genes from mother side, and (**d**) inherited from father.

The gene analysis for the disorder subclass is analyzed in Figure 5. The analysis demonstrates that the diabetes disorder found high occurrence when the maternal and paternal genes have the values 0 or 1 while at the same time it has low chances of Leigh syndrome for all genes. This analysis is based on the 8 disorder sub-class diseases.

**Figure 6:**
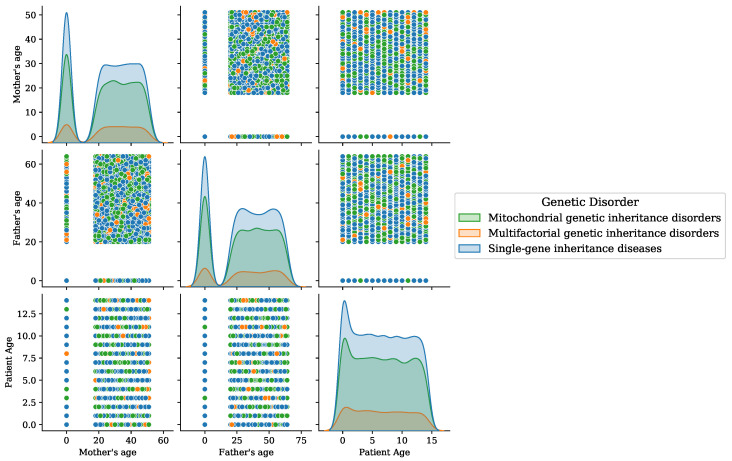
Age analysis of patients for the disorder category.

The age factor of patients is analyzed by the genetic disorder category and is visualized in Figure 6. The age of the mother, father, and patient is examined in this analysis. The analysis demonstrates that there are high chances of genetic disorders when the mother’s age is between 20 and 60 years. When the mother’s age is less than 20 years, the probability of a genetic disorder is low. A high chance of genetic disorder is associated with the age of the father begins between 20 and 70 years. By analyzing the patient’s age, the genetic disorder diseases occur within the 15 years. This analysis shows that age is an important factor that can be used to study genetic disorders.

### 3.3. Data Normalization and Feature Engineering

Feature engineering is a crucial process for machine learning models [40]. Feature engineering techniques are applied to encode data and map data for the genomes dataset. The best fit optimal features are selected for learning models to train and test. For this purpose, important features are selected and unimportant and irrelevant features are dropped. In the current dataset, several features do not contribute to gene disorder prediction and can be dropped to reduce the feature space which improves both the computational complexity and performance of the models.

**Figure 7:**
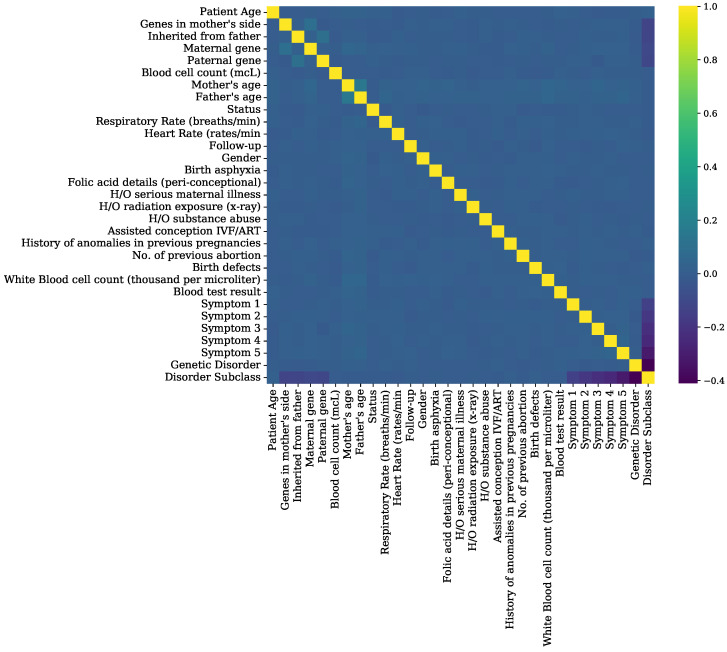
Feature correlation analysis graphs of genomes data.

Feature importance is determined using the DT model and feature correlation is shown in Figure 7. Irrelevant features or features with low importance are not considered for experiments. The features ‘patient Id’, ‘patient first name’, ‘family name’, ‘father’s name’, ‘institute name’, ‘location of institute’, ‘place of birth’, and ‘parental consent’ are dropped due to their low or no contribution in predicting the genetic disorders. The data features ‘test 1’, ‘test 2’, ‘test 3’, ‘test 5’, and ‘autopsy shows birth defect (if applicable)’ are dropped due to lower feature importance values.

The null values are filled with zeros in the dataset. The selected features are encoded with proper categorical data values. The features ‘genes in mother’s side’, ‘inherited from father’, ‘maternal gene’, ’paternal gene’, ‘assisted conception IVF/ART’, ‘history of anomalies in previous pregnancies’, ‘folic acid details (peri-conceptional)’, and ‘H/O serious maternal illness’ contain the values ‘Yes’ and ‘No’ and are mapped by the values 1, and 0, respectively. The features ‘H/O radiation exposure (X-ray)’ and ‘H/O substance abuse’ contain the values of ‘Yes’, ‘No’, and ‘Not applicable’ and are mapped by the values 1, 0, and −1, respectively. The feature ‘status’ contains the values of ‘deceased’ and ‘alive’ that are mapped with 0 and 1, respectively. The feature ‘respiratory rate (breaths/min)’ contains the values of ‘normal (30–60)’ and ‘Tachypnea’ which are mapped with 0 and 1, respectively. The feature ‘heart rate (rates/min’ contains the values of ‘normal’ and ‘Tachypnea’ and are replaced with 0 and 1, respectively. The feature ‘follow-up’ contains the values of ‘Low’ and ‘High’ that are mapped with 0 and 1, respectively. The feature ‘gender’ has ‘male’ and ‘female’ and ‘ambiguous’ and are replaced with 0, 1, and 2, respectively. The feature ‘birth asphyxia’ contains the values of ‘No record’, ‘Not available’, ‘No’, and ‘Yes’ which are replaced with 0, 0, 0, and 1, respectively. The feature ‘birth defects’ contains the values of ‘singular’ and ‘multiple’ that are mapped with 0, and 1, respectively. The feature ‘blood test result’ contains the values of ‘normal’ and ‘abnormal’ and are replaced with 0, and 1, respectively.

### 3.4. Data Balancing

The dataset balancing is applied to achieve high accuracy results from the applied learning techniques [41]. By applying the data balancing approach, the learning models are trained on an equal number of data samples, resulting in efficient results. Before applying the data balancing, the mitochondrial genetic inheritance disorders, multifactorial genetic inheritance disorders, and single-gene inheritance classes have the 10,202, 2071, and 7664 data samples, respectively. We have balanced the dataset by randomly dropping other class data samples by the lowest class count.

### 3.5. Data Splitting

The data splitting is applied to split the data into training and test sets. The data splitting reduces the learning model overfitting and makes the model generalized. For experiments, the dataset is split into different split ratios as the cross-validation to validate the performance of machine learning techniques. The split ratios 0.7:0.3, 0.8:0.2, 0.85:0.15, and 0.9:0.1 are utilized for genomes dataset. These split ratios are used to determine the suitable split for achieving the best learning model.

### 3.6. Applied Learning Techniques

Several machine learning models are applied to analyze the performance of the proposed feature engineering approach. Eight well-known machine learning models, which are reported to show good performance for tasks similar to genetic disorder prediction, are utilized. A brief overview of each of these models is provided here regarding architecture and working mechanisms.

DTC is a supervised machine learning algorithm used for the classification tasks [42]. DTC has a tree-like structure and contains nodes and leaves. The inner nodes contain the data attributes and split them. The outcome label is placed on leaf nodes. The root node in DTC is the highest. DTC algorithms construct decision trees from input data automatically. The goal of the DTC technique is to identify the optimal decision tree by reducing the generalization error. The data attribute selection is a major challenge in DTC. The two data measures information gain and the Gini index are widely utilized. Information gain calculates the change in entropy and can be calculated as

(1) $$Gain\left(S,A\right)=Entropy\left(S\right)-\sum_{\upsilon \epsilon Values(A)}\frac{∥S_{\upsilon }∥}{∥S∥}.Entropy\left(S_{\upsilon }\right)$$

where *S* represents a set of instances and attributes are indicated by *A*.

*Gini index* is used to measure the randomly chosen attributes that would be incorrectly identified. The attribute with a lower *Gini index* is preferred. It is calculated using

(2) $$GiniIndex=1-\sum_{j}p_{j}^{2}$$

RFC is a supervised machine learning model based on multiple decision trees [43]. RFC takes predictions from multiple trees and the final prediction is selected based on majority voting known as the bagging approach [44]. RFC randomly chooses observations to build decision trees and majority voting is taken as the final prediction. RFC is an ensemble learning technique and shows better results than individual classifiers. It reduces the overfitting problems and improves classification performance.

ETC is another ensemble-based, bagged decision tree technique similar to RFC [45]. It uses the same parameter used by the RFC, yet proves to be superior to RFC as it reduces the model variance. The key difference between ETC and RFC is the building of a tree where ETC follows a random split selection of values and not the bootstrap observations [46]. ETC utilizes a meta estimator that fits randomized decision trees on a sample dataset which results in improved accuracy and reduced overfitting.

LR is a supervised statistical learning method used for classifications [47]. For multi-label classification, the ordinal type of LR is utilized. LR predicts the dependent categorical variable using the independent variables. The Sigmoid function is used to map the predicted output to probabilities. The probability is defined by the threshold value. It can be expressed as

(3) $$\log\left[\frac{y}{1-y}\right]=b_{0}+b_{1}x_{1}+b_{2}x_{2}+b_{3}x_{3}+\ldots +b_{n}x_{n}$$

MLP is a classification algorithm that uses a feedforward neural network [48]. MLP consists of multiple fully connected layers. The nodes of the model layers are called neurons. The training process of MLP is iterative and stochastic gradient descent is utilized to optimize the loss function. The output of the layer is dependent on neurons and the neural network weights. Contrary to several complex models [49], MLP has shown superior performance for several tasks.

KNN is a non-parametric supervised learning technique [50] which uses data from nearest neighbors to predict the class of data. KNN works to group the data based on similar points near each other. The classification is performed based on the similarity of data points. The training time is slow due to lazy learning. The data point’s similarity is calculated by using Euclidean distance or similar other distance metrics [51].

XGB utilizes the boosting techniques used for the classification task [52]. XGB is flexible, efficient, and portable. It is based on the parallel gradient boosting tree technique to solve classification problems. To reduce overfitting, XGB uses a better regularization technique. Prediction from the XGB can be made using

(4) F2(x)=σ(0+1∗h1(x)+1∗h2(x))

SVC is a supervised learning algorithm and is mostly used for classification tasks [53]. SVM finds the best fit or optimal hyperplane that separates input data points into two or more components by maximizing the margin between different class samples. The data points on the sides of the classification line represent the categories. The data points are represented by the support vectors. The predictions by using the hyperplane are calculated by the hypothesis function *h* which can be represented as

(5) $$h\left(x_{i}\right)=\left\{\begin{array}{cc} +1 & if\;w.x+b\geq 0\\ -1 & if\;w.x+b\leq 0 \end{array}\right.$$

The applied machine learning approaches are fully hyperparameterized. The iterative process of tuning for employed machine learning models is performed to find the best fit model parameters. The selected parameters help to find efficient accuracy scores. A complete list of hyperparameters for models is provided in Table 3.

### 3.7. Multi-Label Multi-Class Chain Classifier Approach

**Figure 8:**
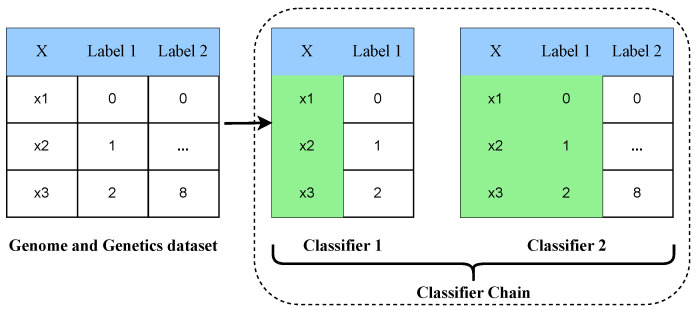
The architectural analysis of the multi-label multi-class classifier chain approach.

The genomes and genetics datasets that are utilized in this study are multi-label multi-class data. We solve the multi-label multi-class classification of genetic disorders and types of disorders as a subclass. For this purpose, the classifier chain approach is utilized for building multi-label multi-class machine learning techniques. The classifier preserves the label correlations in the dataset during the training and testing of models. A connected chain of multiple classifiers is created for a machine learning model. In the classifier chain technique, each model predicts in the order specified by the chain, and the earlier predictions of models in the chain are incorporated by the next models [54,55]. The classifier chain technique uses a chain of classifiers where each classifier uses all the previous classifier’s predictions as input. The total number of classifiers in the classifier chain is equal to the number of classes in the dataset used in this study [56]. The architectural analysis of the classifier chain approach is examined in Figure 8. The macro accuracy, *α*-evaluation score, and Hamming loss are the evaluation metrics that are used for multi-label multi-class data [57,58].

### 3.8. Novel Proposed ETRF Feature Engineering Approach

**Figure 9:**
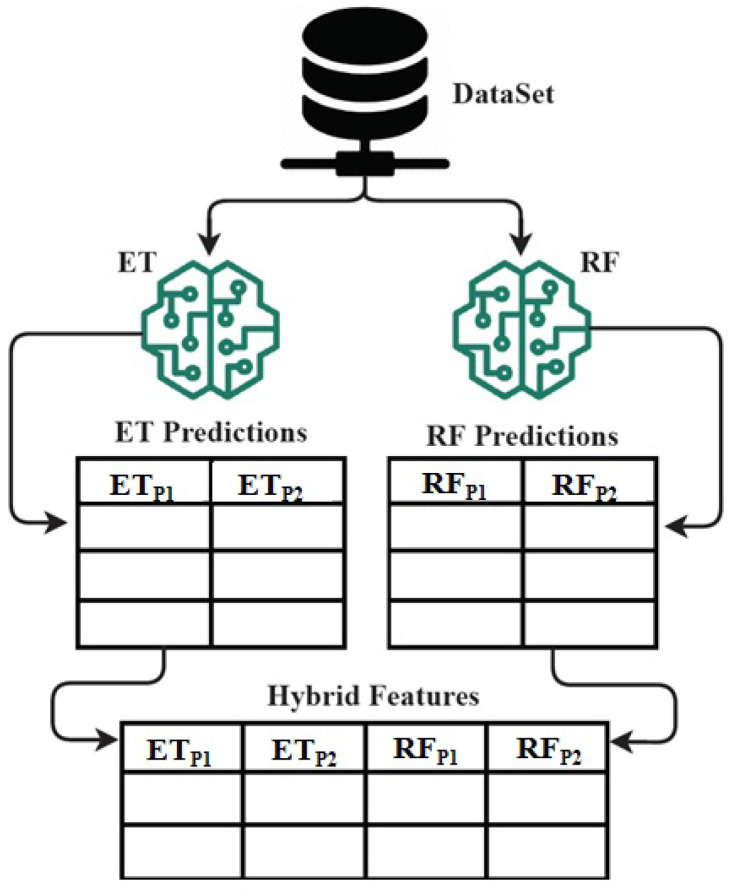
The architecture analysis of proposed ETRF technique for hybrid feature set formation mechanism.

The novel ETRF technique is analyzed in this section. The ETRF approach is formed by combining the ET and RF algorithms. In this research, the ETRF technique is used as a feature extraction technique for learning model building and predicting genetic disorders [59]. The feature set formation and extraction mechanism from the genomes dataset by using the proposed ETRF technique is visualized in Figure 9. The architectural analysis shows that the genomes data samples are input to the ET and RF algorithms separately. The class predicted probabilities are extracted from the RF and ET techniques. A hybrid feature set is formed by combining the extracted class predicted probabilities. The hybrid feature set is later used as an input to applied learning techniques for predicting the genetic disorder and types of disorder.

## 4. Results and Evaluations

### 4.1. Experimental Setup

Results and evaluations of the proposed research approach are examined in this section. Experiments are performed on an Intel I5-8265U CPU, 12GB random access memory (RAM), and an NVIDIA Tesla K80 graphic card. Python and Scikit-learn tools [60] are utilized for building machine learning models. The machine learning models are utilized to predict the genetic disorders and types of genetic disorders.

### 4.2. Evaluation Metrics

The macro accuracy, *α*-evaluation score, recall, precision, Hamming loss, and F1 score are used as evaluation metrics. The followings are the important factors used in evaluation metrics:

- **True Positive**: the number of correctly classified positive samples by the model.
- **True Negative**: the number of correctly classified negative samples by the model.
- **False Positive**: the number of incorrectly classified negative samples by the model as positive.
- **False Negative**: the number of incorrectly classified positive samples by the model as negative.

For multi-label problems, the label-based metrics are evaluated for each label and then averaged over all labels. The macro accuracy metric is computed on individual class labels and then averaged over all classes. The mathematical notations used to calculate the multi-label macro accuracy are expressed here.

(6) $$\lambda -accuracy\;\left(A_{macro}^{j}\right)=\frac{\sum_{j=1}^{n}\left[\left(y_{j}^{\left(i\right)}\wedge {\hat{y}}_{j}^{\left(i\right)}\right)\right]}{\sum_{j=1}^{n}\left[\left(y_{j}^{\left(i\right)}\vee {\hat{y}}_{j}^{\left(i\right)}\right)\right]}$$

where *n* is training instances, $y{\left(i\right)}_{i}$ is the true label, and y(i) is the predicted label.

The hamming loss calculates the proportion of incorrectly predicted target labels to the total number of labels. The number of FN and FP per instance is computed and then averaged over the total number of instances for multi-label classification. The mathematical expression of Hamming loss is given as

(7) $$hamming\;loss=\frac{1}{nL}\sum_{i=1}^{n}\sum_{j=1}^{L}\left[I\left(y_{j}^{\left(i\right)}\neq {\hat{y}}_{j}^{\left(i\right)}\right)\right]$$

where *n* is training instances, $y_{i}^{\left(i\right)}$ is the true label, and ${\hat{y}}_{i}^{\left(i\right)}$ is the predicted label.

For evaluating each multi-label prediction, the *α*-evaluation score is used as the generalized version of Jaccard similarity. The *α*-evaluation score provides the best way to evaluate the multi-label classification results of a learning approach. The mathematical notations for the *α*-evaluation score are expressed as

(8) $$\begin{array}{c} \alpha -evaluation\;score={\left(\frac{\beta M_{x}+\gamma F_{x}}{Y_{x}\vee P_{x}}\right)}^{a}\\ \left(\alpha \geq 0,0\leq \beta ,\gamma \leq 1,\beta =1\right) \end{array}$$

where $M_{x}$ is the number of FNs, $F_{x}$ is the number of FPs, $Y_{x}$ is the combination of TPs and FNs, and $P_{x}$ is the combination of TPs and FPs.

Precision and recall are also utilized as evaluation metrics. Precision calculates the predicted number of samples that correctly belong to the positive class. The recall calculates the predicted number of positive samples out of all positive data. The mathematical notations for expressing the precision and recall are given as

(9) $$\mathrm{Precision}=\frac{\mathrm{True}\;\mathrm{Positive}}{\mathrm{True}\;\mathrm{Positive}+\mathrm{False}\;\mathrm{Positive}}$$

(10) $$\mathrm{Recall}=\frac{\mathrm{True}\;\mathrm{Positive}}{\mathrm{True}\;\mathrm{Positive}+\mathrm{False}\;\mathrm{Negative}}$$

F1 score is based on the combination of precision and recall scores to measure model performance. F1 score is the harmonic mean of precision and recall scores and is calculated as

(11) $$F1\;\mathrm{score}=2*\frac{\mathrm{Precision}*\mathrm{Recall}}{\mathrm{Precision}+\mathrm{Recall}}$$

**Figure 10:**
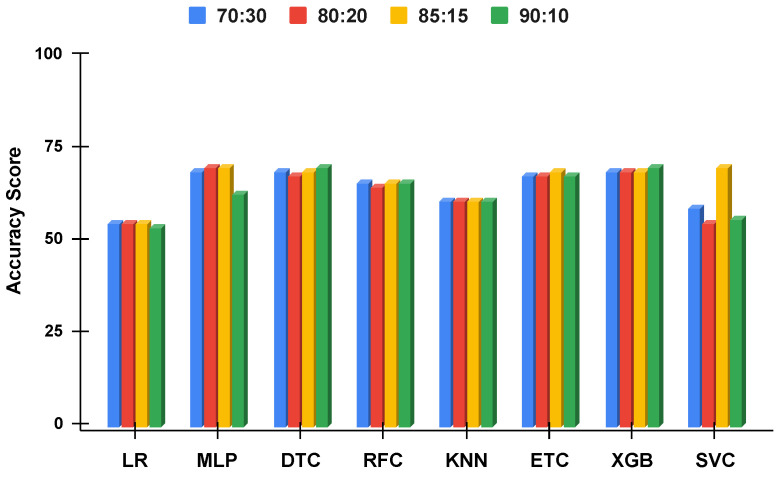
The applied techniques performance comparative analysis of different data split ratios without proposed technique using imbalanced data.

**Figure 11:**
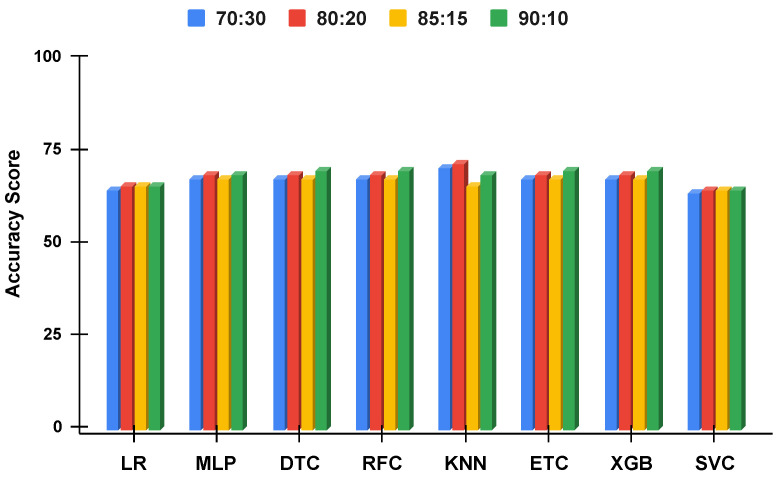
The applied techniques performance comparative analysis of different data split ratios with proposed technique using imbalanced data.

**Figure 12:**
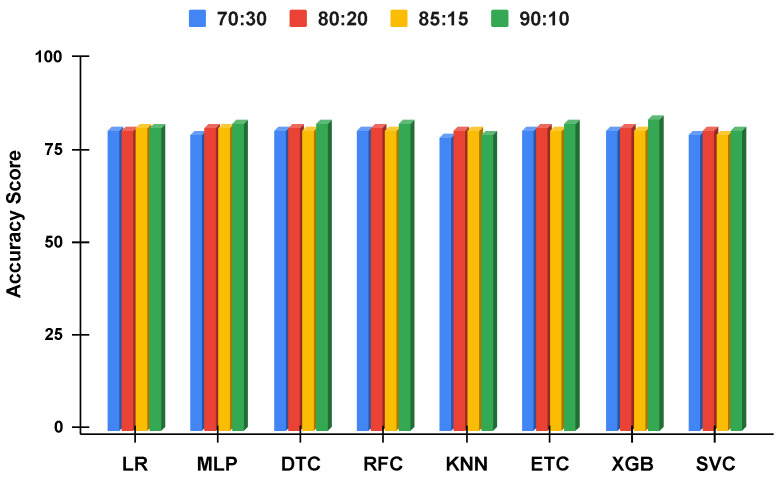
The applied techniques performance comparative analysis of different data split ratios with proposed technique using balanced data.

**Figure 13:**
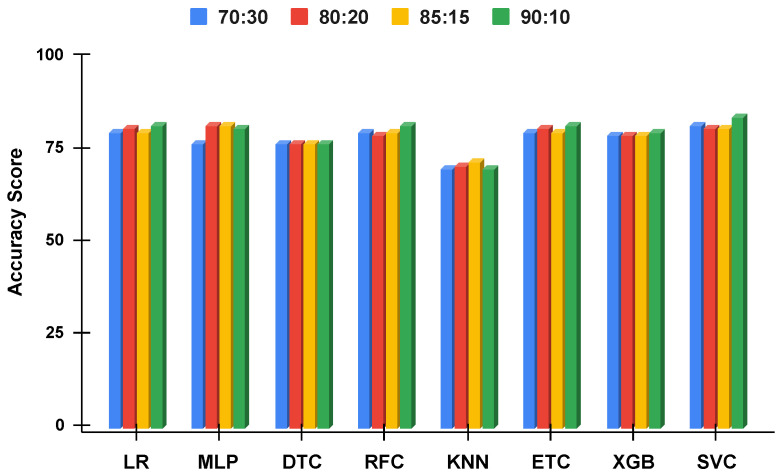
The applied techniques performance comparative analysis of different data split ratios without proposed technique using balanced data.

The accuracy score comparative analysis of applied machine learning techniques with a split of 70:30, 80:20, 85:15, and 90:10 is visualized. The imbalanced dataset accuracy comparative analysis results with and without using the proposed approach are examined in Figure 10 and Figure 11. By applying the data balancing, the comparative analysis of accuracy results with and without using the proposed approach is examined in Figure 12 and Figure 13. This analysis demonstrates the significance of our proposed approach by increasing the accuracy of the results.

### 4.3. Experimental Results with Imbalanced Dataset

The applied machine learning techniques are comparatively evaluated with the imbalanced dataset. For evaluating the performance with different train–test splits, the ratio is varied as 0.7:0.3, 0.8:0.2, and 0.9:0.1.

#### 4.3.1. Results Using 70:30 Split

Table 4 demonstrates the comparative results analysis of machine learning models with and without using the proposed approach. The performance metrics precision, recall, F1 score, and accuracy scores are examined label-wise. The comparative analysis shows that the performance of the models is increased by using the proposed technique. ETC achieves a 59% accuracy score, 54% precision score, 48% recall score, and 49% F1 score for label 1. By using the proposed technique, its performance is elevated to 66% accuracy, 74% precision score, 72% recall score, and 71% F1 for label 1. In the same way, the performance metric scores are improved for label 2. All metrics scores are increased by using the proposed technique.

The multi-label multi-class performance evaluation parameters are also analyzed with a data split of 70:30 Table 5. The performance metrics used are training time (seconds), macro accuracy, hamming loss, and *α*-evaluation score. The comparative results are examined with and without using the proposed technique. The analysis demonstrates that by using the proposed approach, the performance metrics results are increased. SVC model achieves a 59% of accuracy score and by using the proposed approach, its accuracy is increased to 64%. The hamming loss is decreased from 0.24 to 0.18 and the *α*-evaluation score is increased from 86% to 91%. This shows that the proposed approach proves very effective to achieve higher results.

#### 4.3.2. Results Using 80:20 Train–Test Split

Performance of machine learning techniques is analyzed using an 80:20 split as well. The performance results of models with and without using the proposed approach for label 1 and label 2 are examined in Table 6.

The analysis demonstrates that performance metric results are increased by using the proposed approach. MLP achieves a 60% accuracy, 54% precision, 50% recall, and 51% F score for label 1. By using the proposed technique, its performance is significantly improved and it obtains 67%, 75%, 73%, and 72% for accuracy, precision, recall, and F1 scores. Another important point is the increase in the performance of models with a change in train–test split ratios. For example, the accuracies of MLP is increased from 66% to 67%, DTC from 66% to 67%, RFC from 66% to 67%, KNN from 53% to 55%, ETC from 66% to 67%, XGB from 66% to 67%, and SVC from 64% to 65%. An increase in the training data size provides more samples for training which results in improved accuracy.

The multi-label multi-class performance comparative analysis with data split of 80:20 by using the imbalanced dataset is examined in Table 7. Analysis reveals that the performance of the models is increased when used with the proposed features. For example, the macro accuracy score of DTC is increased from 68% to 69% and the *α*-evaluation score from 83% to 90%. The hamming loss is decreased from 0.22 to 0.16. Similarly, the performance of other techniques is also improved when using the proposed approach.

#### 4.3.3. Results Using 85:15 Split Ratio

Table 8 shows the comparative analysis of all the models using the 85:15 train–test split. MLP achieves a 61% accuracy, 55% precision, 53% recall, and 53% F1 score which is the best performance without using the proposed approach. However, when the proposed feature engineering approach is used, the performance of MLP is elevated to 66% accuracy, 74% precision, 72% score, and 71% F1. The performance results for label 2 are also increased. This analysis demonstrates that the results of all metrics are improved by utilizing the proposed approach. Other than that, the performance of models is also increased due to an increase in the size of training data.

The multi-label multi-class comparative analysis of applied learning techniques with and without the proposed approach is also examined in Table 9. Macro accuracy of the KNN model is improved from 61% to 66% using the proposed approach. The hamming loss is decreased from 0.25 to 0.17 and the *α*-evaluation score is increased from 84% to 91%. All other learning techniques results are improvised.

#### 4.3.4. Results Using 90:10 Train–Test Split

In addition to the previous train–test splits, this study utilizes a 90:10 split ratio as well to analyze the performance of models, and the results are given in Table 10. The best performance is obtained using ETC with the proposed approach. ETC achieves 59%, 56%, 48%, and 49% for accuracy, precision, recall, and F1 score which are further improved when the proposed approach is used. Accuracy, precision, recall, and F1 scores are improved to 67%, 76%, 73%, and 72%, respectively. The same is true for label 2 scores. In addition, an increase in performance is also observed due to a change in the size of the training data.

The multi-label multi-class performance comparative analysis with an imbalanced dataset using a data split of 90:10 is examined in Table 11. The analysis is based on the multi-label multi-class metrics results with and without the proposed technique. The SVC technique obtains better performance and its performance is further improved with the proposed approach as its macro accuracy score is increased from 56% to 65% and the *α*-evaluation score from 90% to 92%. The hamming loss is decreased from 0.23 to 0.17. This analysis shows that our proposed approach helps us to achieve higher accuracy scores.

#### 4.3.5. Performance Comparison of All Split Ratios for Imbalanced Dataset

Figure 10 and Figure 11 summarize the performance of machine learning models without the proposed approach and using the proposed approach, respectively. It can be observed that the performance of all the models is elevated using the proposed approach even when using the imbalanced dataset. Instead of using single features, the proposed approach combined features from ET and RF which are more suitable for training the models. Additionally, the class probabilities from these models are used as features that improve the performance of models. Another important observation is the similar performance of the models when used with the proposed approach. All the models tend to show a similar performance with slight variations.

### 4.4. Experimental Results with Balanced Dataset

Similar to experiments using the imbalanced dataset, experiments using the balanced dataset involve different train–test splits of 70:30, 80:20, 85:15, and 90:10. However, the best results are obtained using 80:20 train–test splits, so we discuss only the best results here.

The balanced dataset performance comparative analysis of learning techniques label-wise is examined in Table 12. MLP shows better performance as compared to other models and its performance is further improved when the proposed approach is used. For example, its accuracy is improved from 58% to 74% while the precision, recall, and F1 scores are improved from 57%, 58%, and 57% to 74%, 74%, and 74%, respectively. These results are for label 1, however, a similar trend is observed for label 2 indicating the effectiveness of the proposed approach.

The multi-label multi-class metrics result with and without the proposed approach by using the balanced dataset is examined in Table 13. The analysis utilizes the 80:20 split size with and without the proposed approach. XGB model obtains the highest macro accuracy of 79% without the proposed approach which is further elevated to 82% when used with the proposed approach. Similarly, its hamming loss is decreased from 0.17 to 0.14 and the *α*-evaluation score is increased from 88% to 89%.

Regarding results for the 85 to 15 train–test split ratio, the performance of XGB is superior with 73% accuracy, 73% precision, 72% recall, and 72% F1 score. For multi-label multi-class performance comparative analysis, DTC obtains a macro accuracy score of 77%, hamming loss of 0.14, and the α-evaluation score of 91%. Using a train–test split of 90:10, XGB models obtain the highest accuracy of 76% using the proposed approach. For multi-label multi-class analysis, XGB has macro accuracy of 84%. hamming loss of 0.12 and the α-evaluation score of 92%.

The best individual performance is obtained by the XGB when used with the proposed ETRF feature engineering, as shown in Figure 12. These figures further show that using the proposed approach, all the models improve their performance and the difference in their performance is reduced. Without using the proposed approach, the performance of the models varies significantly as shown in Figure 13.

### 4.5. Results for High Dimensional Real Genomic Data

We applied the proposed XGB model on multi-omics and high dimensional real genomic datasets taken from [61] to validate the performance of the proposed approach. The dataset is available at [62]. It contains the gene expression levels of 22,284 genes (columns) from 64 samples (rows). Experimental results of the proposed approach are provided in Table 14. The performance analysis of the proposed model demonstrates that a 100% accuracy can be obtained. The scores for accuracy, precision, recall, and F1 metrics are also 100%. The classification report indicates a superior performance of the proposed model on an additional dataset.

### 4.6. Performance in Comparison to Existing Studies

Performance analysis of the proposed approach is desired with existing approaches to show the effectiveness of the current study and highlight its performance within the context of existing literature.

A comparative analysis is shown in Table 15 to demonstrate the importance of the current study. For comparison, the models from the selected studies are implemented on the dataset used in this study to make a fair comparison. Results suggest that the proposed approach is superior in terms of overall performance, as well as computational complexity. Using less training time, it can outperform the existing approaches with high macro accuracy and *α*-evluation score, and low Hamming loss.

## 5. Conclusions and Future Work

Machine learning-based approaches have the need of time to build prediction models for the medical field and have the potential to assist medical experts with timely decisions. The prediction of genetic disorders is very important to reduce the risk of fatal outcomes. This study proposed a novel approach to enhance the performance of predictive models for genetic disorders. Two contributions of the study are the use of hybrid features from ET and RF where the class probabilities from these models are combined to make a feature set that is used to train machine learning models. Secondly, this study utilizes a classifier chain approach where the predictions from the preceding models are utilized by the conceding models. Each model in the chain predicts in a manner of its position in the chain. Extensive experiments are carried out with and without using the balanced dataset for the proposed approach. Results indicate that the proposed ETRF technique produces the best results with the XGB model with a 92% *α*-evaluation score, 84% macro accuracy score, and 0.12 Hamming loss. Results are far better than existing state-of-the-art approaches, regarding both performance and computational complexity. This study considers only machine learning models and performance analysis of deep learning models is left for the future. We also intend to apply transfer learning techniques for multi-label multi-class classification to enhance performance for genetic disorder prediction.

## Figures and Tables

**Table 1:** Summary of genetic disorder-related literature.

Ref.	Year	Approach	Dataset	Accuracy (%)	Aim
[21]	2021	Stacked ML Model	AD genetic data of neuroimaging project (ADNI-1)	93	Classify Alzheimer’s disease type of brain disorders using ML.
[25]	2021	Genetic Disease Analyzer (GDA)	GEO dataset	98	Prediction of complex genes and identify genetic classifications that cause complex diseases.
[34]	2021	XGBoost	Alzheimer’s Disease Neuroimaging Initiatives (ADNI)	64	Alzheimer’s Disease Classification Using Genetic Data
[33]	2020	Machine Learning-based model	Genes data	-	Disease gene prediction using machine learning
[35]	2020	Machine Learning-based model	Microarray gene expression dataset of autism spectrum disorder (ASD)	97	Predicting autism spectrum disorder from associative genetic markers of phenotypic groups.
[36]	2020	Random forest classifier	GWAS and GTEx Portals data	81	Machine Learning-based Model for predicting the effect of the deleterious and neutral variant for Alzheimer’s disease.
[37]	2021	Support vector machine	Molecular-based network and brain connectome data	72	Propose a framework for integrating brain connectome data and molecular-based gene association networks to predict brain disease genes.
[27]	2022	Support vector machine	Multifactorial Genetic Inheritance Disorder multiclass dataset	92	Machine learning approaches were used to predict dementia, cancer, and diabetes.
[28]	2021	Random forest classifier	Metagenomic dataset-based on inflammatory bowel disease multi-omics	90	Inflammatory bowel disease prediction using machine learning techniques.
[29]	2020	Random forest classifier	Genetic SNP mutation dataset	92	Machine learning techniques were utilized to predict the COVID-19 infection and related diseases.
[30]	2020	Gradient boosting classifier	Virtual genetic, clinical test data	83	Prediction of familial hypercholesterolemia genetic disorder using the machine learning techniques.
[31]	2017	DOMINO	Genomic dataset	92	Machine learning-based DOMINO was used to Predict dominant mutations in Genes for Mendelian disorders.

**Table 2:** The genomes dataset features descriptive analysis.

Sr No.	Feature	Count	Data Type	Sr No.	Feature	Count	Data Type
1	Patient Id	31,548	object	23	Follow-up	29,382	object
2	Patient Age	30,121	float64	24	Gender	29,375	object
3	Genes in mother’s side	31,548	object	25	Birth asphyxia	29,409	object
4	Inherited from father	30,691	object	26	Autopsy shows birth defect (if applicable)	30,522	object
5	Maternal gene	25,015	object	27	Place of birth	29,424	object
6	Paternal gene	31,548	object	28	Folic acid details (peri-conceptional)	29,431	object
7	Blood cell count (mcL)	31,548	float64	29	H/O serious maternal illness	29,396	object
8	Patient First Name	31,548	object	30	H/O radiation exposure (X-ray)	29,395	object
9	Family Name	12,540	object	31	H/O substance abuse	29,353	object
10	Father’s name	31,548	object	32	Assisted conception IVF/ART	29,426	object
11	Mother’s age	25,512	float64	33	History of anomalies in previous pregnancies	29,376	object
12	Father’s age	25,562	float64	34	No. of previous abortion	29,386	float64
13	Institute Name	24,406	object	35	Birth defects	29,394	object
14	Location of Institute	31,548	object	36	White Blood cell count (thousand per microliter)	29,400	float64
15	Status	31,548	object	37	Blood test result	29,403	object
16	Respiratory Rate (breaths/min)	26,513	object	38	Symptom 1	29,393	object
17	Heart Rate (rates/min	26,535	object	39	Symptom 2	29,326	object
18	Test 1	29,421	float64	40	Symptom 3	29,447	object
19	Test 2	29,396	float64	41	Symptom 4	29,435	object
20	Test 3	29,401	float64	42	Symptom 5	29,395	object
21	Test 4	29,408	float64	43	Genetic Disorder	19,937	object
22	Test 5	29,378	float64	44	Disorder Subclass	19,915	object

**Table 3:** Configuration of hyperparameters for employed machine learning models.

Technique	Hyperparameters
**ETC**	n_estimators = 300, random_state = 5, max_depth = 300, criterion = “gini”, max_features = “sqrt”, bootstrap = False, oob_score = False, ccp_alpha = 0.0
**SVC**	penalty = ‘l2’, loss = ‘squared_hinge’, tol = 1 × 10^−4^, C = 1.0, multi_class = ‘ovr’, fit_intercept = True, max_iter = 1000
**LR**	penalty =‘l2’, tol = 1 × 10^−4^, C = 1.0, fit_intercept = True, solver = ‘lbfgs’, random_state = None, max_iter = 100, multi_class = ‘auto’
**DTC**	max_depth = 300, criterion = “gini”, splitter = “best”, ccp_alpha = 0.0, random_state = None
**RFC**	max_depth = 300, n_estimatorsint = 100, criterion = “gini”, max_features = “sqrt”, random_state = None, bootstrap = True, ccp_alpha = 0.0
**XGB**	use_label_encoder = False, eval_metric = ‘mlogloss’, max_depth = 300, objective = ‘multi:softprob’
**KNN**	n_neighbors = 5, weights = ‘uniform’, leaf_size = 30, metric = ‘minkowski’, algorithm = ‘auto’, p = 2
**MLP**	hidden_layer_sizes = 100, max_iter = 300, activation = ‘relu’, solver = ‘adam’, alpha = 0.0001, learning_rate = ‘constant’, tol = 1 × 10^−4^, epsilon = 1 × 10^−8^, max_fun = 15000

**Table 4:** Comparative analysis of machine learning models using an unbalanced dataset with a data split of 70:30.

Technique	Label 1				Label 2			
	Results without Proposed Technique							
	Accuracy (%)	Precision (%)	Recall (%)	F1 Score (%)	Accuracy (%)	Precision (%)	Recall (%)	F1 Score (%)
**LR**	53	51	42	36	33	19	17	14
**MLP**	58	52	51	50	35	26	25	24
**DTC**	50	44	44	44	29	23	24	24
**RFC**	58	54	46	46	37	28	22	23
**KNN**	46	36	34	33	21	13	12	12
**ETC**	59	54	48	49	37	40	24	26
**XGB**	57	51	49	49	36	30	25	26
**SVC**	49	48	35	34	27	20	14	12
	**Results with Proposed Technique**							
**LR**	64	73	69	66	43	39	38	36
**MLP**	66	74	72	71	45	58	41	40
**DTC**	66	74	72	71	44	51	41	41
**RFC**	66	74	72	71	44	51	41	41
**KNN**	53	67	67	65	35	38	40	37
**ETC**	66	74	72	71	44	51	41	41
**XGB**	66	74	72	71	44	51	41	41
**SVC**	64	73	69	65	42	35	38	36

**Table 5:** The multi-label multi-class performance comparative analysis with an imbalanced dataset using a data split of 70:30.

Technique	Training Time (s)	Macro Accuracy (%)	Hamming Loss	*α*-Evaluation Score
Results without the proposed technique				
LR	3.20	55	0.22	90
MLP	75.34	69	0.18	88
DTC	0.25	69	0.21	83
RFC	4.04	66	0.18	89
KNN	0.05	61	0.25	84
ETC	02.39	68	0.17	89
XGB	75.94	69	0.18	87
SVC	12.06	59	0.24	86
Results with the proposed technique				
LR	2.05	65	0.18	91
MLP	13.17	68	0.18	90
DTC	0.01	68	0.17	90
RFC	0.79	68	0.17	90
KNN	0.02	71	0.23	78
ETC	2.18	68	0.17	90
XGB	7.49	68	0.17	90
SVC	0.28	64	0.18	91

**Table 6:** Performance analysis of machine learning models using an imbalanced dataset with a data split of 80:20.

Technique	Label 1				Label 2			
	Results without Proposed Technique							
	Accuracy (%)	Precision (%)	Recall (%)	F1 Score (%)	Accuracy (%)	Precision (%)	Recall (%)	F1 Score (%)
**LR**	53	52	42	37	33	19	17	14
**MLP**	60	54	50	51	37	27	24	25
**DTC**	49	43	43	43	29	21	21	21
**RFC**	58	54	45	45	36	27	21	22
**KNN**	47	36	34	34	22	12	12	12
**ETC**	59	55	48	49	37	34	23	25
**XGB**	57	52	48	49	36	32	26	28
**SVC**	50	45	35	32	19	17	16	12
	**Results with Proposed Technique**							
**LR**	65	75	71	68	43	39	39	37
**MLP**	67	75	73	72	45	48	40	39
**DTC**	67	75	73	72	45	55	41	41
**RFC**	67	75	73	72	45	54	41	40
**KNN**	55	69	69	66	37	43	42	40
**ETC**	67	75	73	72	45	55	41	41
**XGB**	67	75	73	72	45	55	41	41
**SVC**	65	75	70	67	43	40	38	36

**Table 7:** The multi-label multi-class performance comparative analysis with an imbalanced dataset using a data split of 80:20.

Technique	Training Time (s)	Macro Accuracy (%)	Hamming Loss	*α*-Evaluation Score
Results without the proposed technique				
LR	3.47	55	0.22	90
MLP	69.57	70	0.17	89
DTC	0.29	68	0.22	83
RFC	4.74	65	0.19	89
KNN	0.03	61	0.24	84
ETC	14.45	68	0.18	89
XGB	91.50	69	0.18	87
SVC	14.04	55	0.24	88
Results with the proposed technique				
LR	2.16	66	0.17	91
MLP	10.95	69	0.16	90
DTC	0.01	69	0.16	90
RFC	0.88	69	0.16	90
KNN	0.03	72	0.26	79
ETC	2.44	69	0.16	90
XGB	9.13	69	0.16	90
SVC	0.34	65	0.17	92

**Table 8:** Performance comparative analysis of models using an imbalanced dataset and 85:15 split.

Technique	Label 1				Label 2			
	Results without Proposed Technique							
	Accuracy (%)	Precision (%)	Recall (%)	F1 Score (%)	Accuracy (%)	Precision (%)	Recall (%)	F1 Score (%)
**LR**	54	52	42	37	32	15	16	13
**MLP**	61	55	53	53	38	30	29	29
**DTC**	51	44	44	44	30	24	25	24
**RFC**	58	54	46	46	36	27	21	22
**KNN**	47	33	33	32	22	13	13	12
**ETC**	59	54	47	48	37	33	23	25
**XGB**	57	51	48	49	35	27	23	24
**SVC**	41	44	34	29	25	20	20	14
	**Results with Proposed Technique**							
**LR**	65	75	70	67	42	40	38	36
**MLP**	66	74	72	71	43	45	40	39
**DTC**	66	74	72	70	42	47	40	40
**RFC**	66	74	72	71	43	48	40	40
**KNN**	65	74	70	68	39	44	38	38
**ETC**	66	74	72	70	42	47	40	40
**XGB**	66	74	72	71	42	45	39	39
**SVC**	64	74	70	66	41	35	38	35

**Table 9:** The multi-label multi-class performance comparative analysis with an imbalanced dataset using a data split of 85:15.

Technique	Training Time (s)	Macro Accuracy (%)	Hamming Loss	*α*-Evaluation Score
Results without the proposed technique				
LR	4.08	55	0.22	91
MLP	104.1	70	0.16	89
DTC	0.31	69	0.21	84
RFC	5.42	66	0.18	89
KNN	0.04	61	0.25	84
ETC	16.8	69	0.18	89
XGB	99.37	69	0.18	87
SVC	16.4	70	0.25	77
Results with the proposed technique				
LR	2.49	66	0.17	92
MLP	13.58	68	0.17	90
DTC	0.01	68	0.17	90
RFC	0.95	68	0.17	90
KNN	0.03	66	0.17	91
ETC	2.55	68	0.17	90
XGB	9.40	68	0.17	90
SVC	0.38	65	0.18	92

**Table 10:** Performance comparative analysis using an imbalanced dataset with a data split of 90:10.

Technique	Label 1				Label 2			
	Results without Proposed Technique							
	Accuracy (%)	Precision (%)	Recall (%)	F1 Score (%)	Accuracy (%)	Precision (%)	Recall (%)	F1 Score (%)
**LR**	53	57	42	37	34	24	17	14
**MLP**	57	56	49	48	36	31	24	24
**DTC**	50	44	45	44	30	23	24	24
**RFC**	58	56	46	47	38	39	23	25
**KNN**	46	34	33	32	22	13	12	12
**ETC**	59	56	48	49	38	40	26	28
**XGB**	57	52	49	50	36	33	26	28
**SVC**	51	40	41	35	31	15	19	15
	**Results with Proposed Technique**							
**LR**	66	77	71	68	43	40	38	37
**MLP**	67	76	73	71	45	45	40	40
**DTC**	67	76	73	72	45	50	41	41
**RFC**	67	76	73	73	45	51	41	41
**KNN**	62	71	71	70	41	50	42	42
**ETC**	67	76	73	72	45	50	41	41
**XGB**	67	76	73	72	45	50	42	42
**SVC**	65	76	70	66	43	41	38	36

**Table 11:** The multi-label multi-class performance comparative analysis using a data split of 90:10.

Technique	Training Time (s)	Macro Accuracy (%)	Hamming Loss	*α*-Evaluation Score
Results without the proposed technique				
LR	5.23	54	0.22	91
MLP	40.5	63	0.19	90
DTC	0.33	70	0.20	84
RFC	5.70	66	0.18	90
KNN	0.04	61	0.25	84
ETC	17.62	68	0.18	89
XGB	106.58	70	0.18	88
SVC	19.0	56	0.23	90
Results with the proposed technique				
LR	2.76	66	0.17	92
MLP	17.6	69	0.16	91
DTC	0.01	70	0.16	90
RFC	1.02	70	0.16	90
KNN	0.03	69	0.19	87
ETC	2.82	70	0.16	90
XGB	15.7	70	0.16	90
SVC	0.37	65	0.17	92

**Table 12:** Comparative analysis of applied machine learning models using a balanced dataset with a data split of 80:20.

Technique	Label 1				Label 2			
	Results without Proposed Technique							
	Accuracy (%)	Precision (%)	Recall (%)	F1 Score (%)	Accuracy (%)	Precision (%)	Recall (%)	F1 Score (%)
**LR**	55	54	56	54	41	18	22	19
**MLP**	58	57	58	57	41	26	24	24
**DTC**	47	47	47	47	32	24	26	25
**RFC**	57	56	57	55	42	23	23	21
**KNN**	37	37	37	36	25	14	14	14
**ETC**	58	57	58	57	43	47	26	26
**XGB**	54	53	54	53	39	41	26	27
**SVC**	37	44	37	027	25	20	20	15
	**Results with Proposed Technique**							
**LR**	72	73	73	72	57	38	41	39
**MLP**	74	74	74	73	51	59	42	41
**DTC**	73	73	73	73	59	50	45	44
**RFC**	73	73	73	73	59	50	44	44
**KNN**	71	71	71	71	56	44	44	44
**ETC**	73	73	73	73	59	50	44	44
**XGB**	73	73	73	73	59	49	44	44
**SVC**	72	73	73	72	38	58	41	39

**Table 13:** The multi-label multi-class performance comparative analysis using balanced data with 80:20 split.

Technique	Training Time (s)	Macro Accuracy (%)	Hamming Loss	*α*-Evaluation Score
Results without the proposed technique				
LR	0.99	81	0.16	87
MLP	17.21	82	0.14	89
DTC	0.08	77	0.19	87
RFC	1.63	79	0.16	89
KNN	0.01	71	0.24	85
ETC	4.54	81	0.14	90
XGB	19.98	79	0.17	88
SVC	3.41	81	0.17	82
Results with the proposed technique				
LR	0.50	81	0.14	91
MLP	6.81	82	0.14	91
DTC	0.01	82	0.14	89
RFC	0.54	82	0.14	89
KNN	0.01	81	0.15	88
ETC	1.32	82	0.14	89
XGB	3.32	82	0.14	89
SVC	0.09	81	0.14	91

**Table 14:** Results for the proposed XGB as classification model for multi-omics and high dimensional real genomic dataset.

Category	Precision	Recall	F1 Score
0	1.00	1.00	1.00
1	1.00	1.00	1.00
2	1.00	1.00	1.00
3	1.00	1.00	1.00
4	1.00	1.00	1.00
Average	1.00	1.00	1.00
Accuracy	1.00		

**Table 15:** Comparative analysis of proposed approach with state-of-the-art approaches.

Reference	Year	Technique	Training Time (s)	Macro Accuracy (%)	Hamming Loss	α-Evaluation Score (%)
[63]	2020	SVM	7.10	73	0.22	88
[64]	2020	KNN	0.01	70	0.25	86
[65]	2020	KNN	0.01	70	0.25	86
[66]	2020	RF	2.48	82	0.14	90
[67]	2021	KNN	0.01	70	0.25	86
**Proposed**	**2022**	**ETRF + XGB**	**3.59**	**84**	**0.12**	**92**

## Data Availability

Not applicable.
